# Whole gel processing procedure for GeLC-MS/MS based proteomics

**DOI:** 10.1186/1477-5956-11-17

**Published:** 2013-04-23

**Authors:** Sander R Piersma, Marc O Warmoes, Meike de Wit, Inge de Reus, Jaco C Knol, Connie R Jiménez

**Affiliations:** 1 Department of Medical Oncology, OncoProteomics Laboratory, VU University Medical Center, Amsterdam, The Netherlands

**Keywords:** In-gel digestion, GeLC-MS/MS, Clinical proteomics

## Abstract

**Background:**

SDS-PAGE followed by in-gel digestion (IGD) is a popular workflow in mass spectrometry-based proteomics. In GeLC-MS/MS, a protein lysate of a biological sample is separated by SDS-PAGE and each gel lane is sliced in 5–20 slices which, after IGD, are analyzed by LC-MS/MS. The database search results for all slices of a biological sample are combined yielding global protein identification and quantification for each sample. In large scale GeLC-MS/MS experiments the manual processing steps including washing, reduction and alkylation become a bottleneck. Here we introduce the whole gel (WG) procedure where, prior to gel slice cutting, the processing steps are carried out on the whole gel.

**Results:**

In two independent experiments human HCT116 cell lysate and mouse tumor tissue lysate were separated by 1D SDS PAGE. In a back to back comparison of the IGD procedure and the WG procedure, both protein identification (>80% overlap) and label-free protein quantitation (R^2^=0.94) are highly similar between procedures. Triplicate analysis of the WG procedure of both HCT116 cell lysate and formalin-fixed paraffin embedded (FFPE) tumor tissue showed identification reproducibility of >88% with a CV<20% on protein quantitation.

**Conclusions:**

The whole gel procedure allows for reproducible large-scale differential GeLC-MS/MS experiments, without a prohibitive amount of manual processing and with similar performance as conventional in-gel digestion. This procedure will especially enable clinical proteomics for which GeLC-MS/MS is a popular workflow and sample numbers are relatively high.

## Background

In this study we focus on streamlining of the workflow for differential analysis of complex protein mixtures by SDS-PAGE, in-gel digestion (IGD) and LC-MS/MS. Typically, an entire gel lane is cut in 5–20 similar slices and after IGD and nanoLC MS/MS the database search results of the individual fractions are combined yielding global protein identification and quantification of the whole complex protein sample, this approach is termed GeLC-MS/MS. We and others [1,2] have shown previously that GeLC MS/MS is a useful approach for differential protein expression profiling [3], biomarker discovery [4,5] and abundant protein depletion method evaluation [6].

In-gel digestion of gel-separated proteins is historically the key procedure for protein identification by mass spectrometry [7] (for protocol see [8]). Advantages of SDS-PAGE include complete protein solubilisation by SDS, high tolerance to salts, buffers and detergents, and consistent digestion by trypsin. The gel electrophoresis step removes detergents, buffers and salts from the protein extract that may interfere with mass spectrometry analysis, and provides a matrix for protein digestion by trypsin. In contrast to peptide separation strategies such as SCX, in SDS-PAGE all tryptic peptides of a protein are retained in a single fraction. Additionally, SDS-PAGE allows for an intermediate level of quality control (Coomassie staining pattern) prior to in-gel digestion, which is important for core labs servicing many collaborators and dealing with a wide range of (clinical) sample types and sample qualities. In the past years solution digestion procedures combined with SCX first-dimension separation have gained popularity for large-scale proteomics experiments [9]. One of the reasons for this development is the labor-intensive nature of in-gel digestion. The bottleneck in scaling up the number of slices is the number of washing, reduction and alkylation steps that need to be performed. This number scales linearly with the number of gel slices. If most process steps could be performed on the whole gel, instead of on each gel slice separately, a considerable reduction in time and labor could be realized.

**Figure 1:**
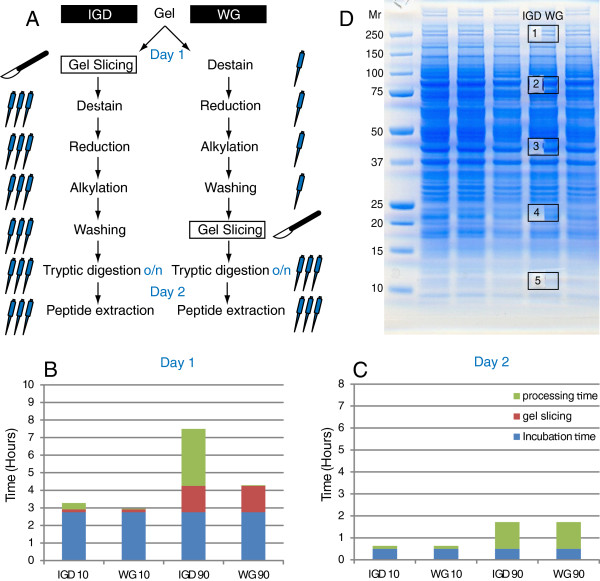
**A schematic overview of the whole-gel procedure (WG) vs. the in-gel digestion procedure (IGD). A**. The number of pipettes next to the workflow indicates the amount of manual processing. **B**. manual processing steps, incubation and gel slicing time breakdown for day 1 for WG and IGD procedures for 1 sample (10 bands) and 9 samples (90 bands). **C** manual processing steps and incubation time breakdown for day 2. Details for Figure 1B and 1C can be found in Additional file 1: Table 1. **D**. Gel image showing the location of gel slices IGD 1–5 processed by in-gel digestion (empty regions) and the corresponding counterpart WG 1–5 region processed by the whole-gel procedure for HCT116 cell lysate.

For this purpose we developed a whole-gel procedure (Figure 1A). Washing, reduction and alkylation steps are performed on the whole gel prior to gel slicing. The gel is sliced just prior to trypsin incubation (overnight in both procedures; o/n) and is guided by pre-stained marker bands and a scanned image of the gel after Coomassie staining.

In small-scale protein identification experiments focusing on one or several selected gelbands IGD is preferred because the Coomassie stain helps in cutting out the selected band(s). In GeLC-MS/MS, however, the whole lane is cut in 5–20 equally sized slices, therefore, gel cutting after destaining but guided by the pre-stained markers is sufficient. We show that the whole-gel procedure yields highly similar protein identification and quantification for complex samples as the conventional in-gel digestion procedure with high reproducibility. Therefore, the whole-gel procedure provides a fast and good alternative to the conventional in-gel digestion protocol in large-scale (clinical) differential proteomics experiments. Moreover, we show that the WG procedure can be applied with similar performance and reproducibility to both cell lysate as well as clinically relevant FFPE material.

## Results and discussion

**Figure 2:**
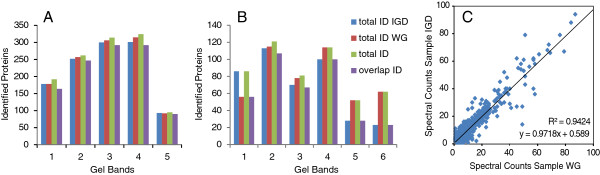
**Protein identification and quantification. A**. Number of protein identifications for each gel slice for IGD (blue), WG (orange), total number of protein IDs in each gel slice (green) and the number of proteins identified in both gel slices (purple) for human HCT116 cell lysate and **B**. for mouse tumor tissue lysate. **C**. Spectral counts of proteins identified in both slice IGD and WG plotted for all 5 slice pairs combined for human CRC cell lysate. The line is a linear fit to the data, the slope and corresponding R^2^ value of the fit are indicated.

In Figure 1A the whole-gel procedure (WG) is compared with the conventional in-gel digestion (IGD) procedure. In this paper the whole gel (WG) procedure refers to the WG workflow in Figure 1A, whereas IGD refers to the in gel digestion workflow in Figure 1A. The conventional protocol starts with gel slicing, this multiplies all downstream volume transfer steps by the number of slices, in addition to the time spent opening and closing lids of reaction tubes. In the whole-gel procedure all washing, reduction and alkylation steps are performed on the intact gel and are thus limited to a single 25 ml addition and removal step for each gel. Only after the final washing step but prior to trypsin incubation, the gel is sliced in the appropriate number of slices (e.g. 10 per lane) and subsequently the in-gel digestion procedure is followed further. The pipet symbol indicates the relative number of pipetting steps in the WG procedure compared to conventional IGD for a large-scale experiment. Obviously, the hands-on time for gel slicing in both WG and IGD procedures is identical (day 1). The same holds for the trypsin incubation step (o/n) and subsequent peptide extraction steps (day 2). However, the main hands-on time is spent on processing steps prior to trypsin incubation; these steps are highly streamlined in the WG procedure. As an example the processing time and incubation times are calculated for processing 10 and 90 gel slices (corresponding to 1 and 9 sample(s), respectively) using the WG and IGD procedures (Figure 1B and C). For 10 gel slices the difference between the two procedures is marginal, while for 90 gel slices the processing time on day one is much higher for the IGD procedure as compared to the WG procedure. It is assumed that for processing 90 slices lab equipment (vortex, heating block, centrifuge, vacuum concentrator) with sufficient capacity is available to accommodate all slices in parallel. In Additional file 1: Table S1 the details for Figure 1B and C can be found. To benchmark the performance of the WG procedure with respect to the conventional gel slicing followed by IGD we designed the following experiment. A 4-12% gradient SDS-PAGE gel was loaded with human HCT116 colorectal cancer (CRC) cell lysate protein aliquots. From a lane in the center of the gel five regions were selected spanning the Mr range from 180 kDa-10 kDa (Figure 1D). Half of the each region was excised carefully with a scalpel (Figure 1D, labeled IGD 1–5) and processed by IGD. After digital scanning, the gel was processed following the WG procedure. Just prior to digestion, the other half of each region (Figure 1D, labeled WG 1–5) was excised. The same solutions were used for both procedures and samples were processed in parallel on the same day. IGD and WG slice pairs were analyzed consecutively by LC-MS/MS to ensure maximal comparability of both workflows. The experiment was repeated independently using mouse tumor tissue lysate separated in a 12% SDS-PAGE gel cut in 6 gel slices as described in the methods section. In Figure 2A (HCT116 cell lysate) and 2B (mouse tumor tissue lysate) the number of protein identifications for IGD and WG are shown, in addition to the total number of identified proteins and the overlap in identified proteins. In Table 1 the corresponding numerical values are shown, in addition to the % overlap in identified proteins and the median theoretical Mr of identified proteins in each gel slice. In Additional file 2: Table S2 and Additional file 3: Table S3 the protein and peptide identifications, respectively, for HCT116 cell lysate can be found. In Additional file 4: Table 4 and Additional file 5: Table S5 the protein and peptide identifications, respectively, for mouse tumor tissue lysate can be found. The ID overlap ranges from 85-95% for the HCT116 cell lysate (4-12% gradient gel) and 83-88% for gel slices 2–4 for the mouse tumor tissue lysate (12% gel). For the mouse tumor tissue lysate in slice 1 more protein IDs are found for the IGD whereas in slices 5 and 6 more IDs are found for WG, in these 3 bands the ID overlap is lower (37-65%), this may be related to the gel used (4–12% gradient vs 12%). Overall, Table 1 indicates highly similar performance of the WG and IGD procedures at the protein identification level. The median calculated Mr for proteins identified in each of the analyzed gel regions is consistent with the Mr marker lane (Figure 1B and Table 1). Similarly, the total number of identified proteins in the analyzed gel regions is roughly proportional to the Coomassie staining intensity. Both observations underscore the consistency between SDS-PAGE and LC-MS/MS protein identification. In addition to protein identification also protein quantification is of key importance in differential protein analysis. For the human cell lysate all proteins identified in both IGD and WG slice pairs (overlap between IGD and WG data sets) were quantified by spectral counting for the five gel regions (N=1085). A highly similar quantitative response is observed for the majority of the identified proteins in the IGD vs. the WG samples. In Figure 2C spectral counts for each protein in the IGD sample vs. the corresponding WG sample is plotted and a highly positive correlation is found (R^2^ 0.94, slope 0.97). Figures 1 and 2 illustrate that the WG procedure performs highly similar as the IGD procedure, both on the qualitative level (protein identification) as well as on the quantitative level (label-free protein quantitation).

**Table 1:** Number of protein identifications using the whole gel (WG) procedure vs in gel digestion (IGD)

**Human HCT116 cell lysate**						**Mouse tumor tissue lysate**					
Gel Band	1	2	3	4	5	1	2	3	4	5	6
Total ID IGD	178	252	300	301	93	86	113	70	100	28	23
Total ID WG	178	257	306	315	92	56	115	78	114	52	62
Total ID	192	262	314	324	95	86	121	81	114	52	62
Overlap ID	164	247	292	292	90	56	107	67	100	28	23
Only IGD	14	5	8	9	3	30	6	3	0	0	0
Only WG	14	10	14	23	2	0	8	11	14	24	39
Overlap*	85%	94%	93%	90%	95%	65%	88%	83%	88%	54%	37%
Median Mr (kDa)	176	83	44	23	12	124	69	38	29.5	19	15

*Overlap =(Overlap ID/total ID)*100%.

**Figure 3:**
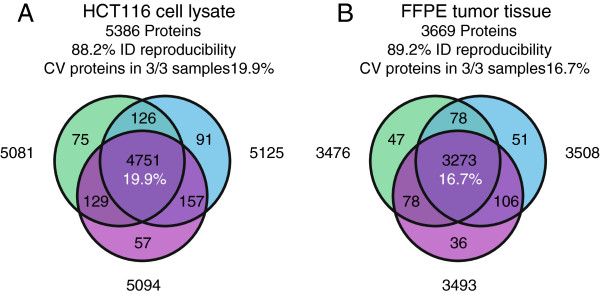
**Workflow reproducibility of the whole gel procedure. A**. Venn diagram of workflow triplicate WG procedure for HCT116 cell lysate. **B** Venn diagram of workflow triplicate WG procedure for FFPE tumor tissue material. In black: number of identified proteins, in white (central overlap): coefficient of variance on spectral counts of proteins identified in 3/3 samples.

Finally, to assess workflow reproducibility, HCT 116 cell lysate and an FFPE tumor tissue sample were separated on three separate SDS-PAGE gradient gels. Gels were processed separately in parallel by the WG procedure. Entire lanes were cut in 5 slices per sample and digested. The 3 × 5 HCT116 fractions yielded in total 5386 proteins with 5081–5125 proteins identified in each individual sample, and 88.2% overlap in protein ID’s between the three gels (Figure 3). The coefficient of variance (CV) on spectral counts for proteins identified in 3/3 replicates was 19.9% indicating good reproducibility of protein quantitation. The challenging FFPE tumor tissue samples yielded in total 3669 proteins with 3476–3508 proteins identified in each sample, with 89.2% overlap in protein ID’s and a CV of 16.6%. These distinctly different biological samples show highly similar workflow reproducibility metrics for protein ID (overlap in protein IDs) and protein quantitation (%CV on spectral counts). The difference in depth of proteome coverage reflects the origin of the material analyzed: fresh cancer cell lysate vs cross-linked, archived patient tumor tissue sections. In Additional file 6: Table S6 and Additional 7: Table S7 the protein and peptide identifications, respectively, for HCT116 cell lysate can be found. In Additional file 8: Table S8 and Additional file 9: Table S9 the protein and peptide identifications, respectively, for FFPE tumor tissue can be found.

The key advantage of the whole-gel procedure over the conventional in-gel digestion procedure for large scale differential proteomics experiments is the reduction of the number of manual processing steps. Thus, washing, reduction and alkylation on the intact gel is as efficient as for individual gel slices. DTT and iodoacetamide concentrations do not need to be adjusted and also incubation time does not have to be extended. Care has to be taken to shake the gel gently on a rocking platform instead of a vortex used for the cut-out gel cubes. Diffusion via the surface of the gel is sufficient to allow equilibration with the solutions in the time indicated. Fading of the Coomassie staining level during the whole-gel procedure is comparable to that observed for individual gel cubes indicating similar equilibration and washing efficiency. Even after washing, reduction and alkylation sufficient staining intensity of the pre-stained marker bands remains to confidently and reproducibly slice the gel in the WG procedure, especially when combined with a digital scan after Coomassie staining prior WG processing. Having established that the whole gel procedure performs similar to conventional in-gel digestion procedure, we have applied the method to numerous differential experiments of cancer cell lines and tissues with up to 30 samples (300 gel slices) now being routine (to be published elsewhere). Alternative strategies to process large numbers of IGD samples have been reported using 96-well plates [10] or 96-well-filter plates [11,12] where all solvent manipulation steps are performed by multichannel pipette or by centrifugation, respectively. We envision a combination of the WG procedure for upfront SDS-PAGE gel clean-up followed by gel slicing and slice processing in 96 well filter plates for protein digestion and peptide extraction steps.

## Conclusions

The whole gel procedure retains the advantages of GeLC-MS/MS while minimizing the prohibitive number of manual steps in large-scale experiments and is a viable alternative to solution digestion based SCX workflows for large-scale quantitative proteomics experiments.

## Materials and methods

### Chemicals

Acetonitrile, methanol and formic acid were purchased from Biosolve (Biosolve, Valkenswaard, The Netherlands), LDS (lithium dodecyl sulfate) sample buffer and pre-casted gradient gels were purchased from Invitrogen (Invitrogen Carlsbad, CA), dithiotreitol, phosphoric acid (85%), NH_4_SO_4,_ NH_4_HCO_3_ and iodoacetamide were purchased from Sigma (Sigma-Aldrich, St Louis, MO) and sequence-grade trypsin was purchased from Promega (Promega, Madison, WI).

### Preparation of lysates and electrophoresis

HCT116 colon cancer cell line lysate was made by washing a 70% confluent T75 flask 3 times with 1 ml PBS. To the adhering cells 1 ml reducing LDS sample buffer was added and cells were scraped from the flask and transferred to a 1.5 ml reaction tube. After boiling the protein extract for 5 min and cooling-down on ice the denatured and reduced lysates were centrifuged at 16.000 RCF for 10 minutes and 15 μl of the clear supernatant (approximately 40 μg protein) was applied to a 1 mm 4-12% reducing NuPage gel and run for 45 min at 200 V. Mouse breast tumor tissue (BRCA^−/−^ P53 ^−/−^) (20 mg) was manually disintegrated in 800 μl SDS sample buffer (62.5 mM Tris–HCl, 2% w/v SDS, 10% v/v glycerol, and 0.0025% bromophenol blue, 100 mM DTT, pH 6.8) using a pellet pestle microgrinder as described previously [5]. After boiling the protein extract for 5 min and cooling-down on ice the denatured and reduced lysates were centrifuged at 16.000 RCF for 15 minutes and approximately 50 μg protein was applied to a home-made 12% acrylamide SDS page gel (BioRad, Hercules, CA) and run for 45 min at 200 V. For the workflow reproducibility experiment a FFPE carcinoma sample collected from the tissue archive of the department of pathology at the VU University medical center (VUmc), Amsterdam, the Netherlands was used in compliance with the institution’s ethical regulations. An area of at least 70% tumor cells was demarcated on a 4-μm haematoxylin- and eosin-stained tissue section. Adjacent serial sections of 10μm were cut and following deparaffination and staining with haematoxylin the tumor tissue was macrodissected using a scalpel. The macrodissected tissue particles were taken up into reducing LDS sample buffer (1:10 w:v) and the mixture was boiled for 1 hour. The denatured and reduced FFPE lysate was centrifuged at 16.000 RCF for 10 minutes and 30 μl of the clear supernatant (approximately 50 μg protein) was applied next to 50 μg of HCT 116 lysate on a 1.5 mm 4-12% reducing NuPage gel. Three gels were used containing an FFPE lane and an HCT116 lane.

SDS-PAGE gels were washed briefly with milliQ water and were subsequently fixed in 50% EtOH/1% phosphoric acid for 15 min. After washing with MilliQ water, the gels were stained overnight with 1% Coomassie brilliant blue R250 in 40% MeOH/1% phosphoric acid containing 1.5 M NH_4_SO_4_. After staining, the gels were washed with milliQ water to remove background Coomassie stain and were scanned using a digital scanner (Hewlett-Packard, Palo Alto, CA).

### In-gel digestion (IGD) procedure

Gel processing and in-gel digestion was performed in a keratin-free laminar flow cabinet. Gel slices were cut from the Coomassie-stained and washed SDS-PAGE gel on a clean glass plate and were further diced in 1 mm^3^ cubes using a scalpel and were transferred to a 1.5 ml reaction tube for further processing. Gel cubes were vortexed in 400 μl 50 mM NH_4_HCO_3_ for 10 min. Supernatant was removed and the gel cubes were vortexed in 400 μl 50 mM NH_4_HCO_3_/50% acetonitrile for 10 min. After removal of the supernatant this wash-step was repeated once. Gel cubes were reduced in 10 mM DTT in 50 mM NH_4_HCO_3_ at 56°C in a heating block for 60 min. After cooling down the supernatant was removed and gel cubes were alkylated with 54 mM iodoacetamide in 50 mM NH_4_HCO_3_ for 45 min in the dark at RT. Supernatant was removed and gel cubes were vortexed in 400 μl 50 mM NH_4_HCO_3_ for 10 min. supernatant was removed and the gel cubes were vortexed in 50 mM NH_4_HCO_3_/50% acetonitrile. After removal of the supernatant this wash-step was repeated once. Supernatant was removed and gel cubes were dried in a vacuum centrifuge at 50°C for 10 min. Dried gel slices were covered with trypsin solution (6.25 ng/μl in 50 mM NH_4_HCO_3_), after rehydration of the cubes excess trypsin was removed and gel cubes were covered with 50 mM NH_4_HCO_3_. Digestion proceeded overnight at 25°C in a heating block. After digestion, peptides were extracted from the gel cubes with 100 μl 1% formic acid by vortexing. Supernatant was transferred to a 1.5 ml tube and gel cubes were extracted with 100 μl 5% formic acid/50% acetonitrile. Supernatant was pooled with the first eluate. The second extraction was repeated once and the supernatant was pooled with previous extracts. Extracts were stored at −20°C. Prior to LC-MS extracts were concentrated in a vacuum centrifuge at 50°C and volumes were adjusted to 50 μl by adding 0.05% formic acid.

### Whole-gel (WG) procedure

The Coomassie-stained and washed SDS-PAGE gel was transferred to a closed container of similar dimensions (w × l) as the gel and was washed by gentle shaking in 25 ml 50 mM NH_4_HCO_3_ for 10 min. Supernatant was removed and the gel was washed in 50 mM NH_4_HCO_3_/50% acetonitrile for 10 min. After removal of the supernatant this wash-step was repeated once. The entire gel was reduced in 25 ml 10 mM DTT in 50 mM NH_4_HCO_3_ at 56°C in a closed water bath for 60 min. After cooling down the supernatant was removed and the gel was alkylated with 25 ml 54 mM iodoacetamide in 50 mM NH_4_HCO_3_ for 45 min. in the dark at RT. Supernatant was removed and the gel was washed in 25 ml 50 mM NH_4_HCO_3_ for 10 min. Supernatant was removed and the gel was washed in 25 ml 50 mM NH_4_HCO_3_/50% acetonitrile. After removal of the supernatant this wash-step was repeated once. The gel was transferred to a clean glass plate in a laminary flow cabinet and based on the previously scanned image, and using the prestained markers as a guide, gel slices were cut from the gel. Gel slices were further diced in 1 mm^3^ cubes and transferred to a 1.5 ml reaction tube. For a whole lane, the gel is typically cut in 5–20 equal-sized gel slices. Gel cube drying, tryptic digestion, peptide extraction and volume adjustion was identical to the in-gel digestion procedure. For the WG workflow reproducibility analysis the three gels were processed in separate containers and the gel lanes were sliced in 5 slices per lane. Starting from the same HCT116 and FFPE tumor tissue sample, the three gels were processed and measured in parallel. Therefore, the reproducibility shown reflects reproducibility of the entire workflow.

### LC-MS/MS

Peptides were separated by an Ultimate 3000 nanoLC-MS/MS system (Dionex LC-Packings, Amsterdam, The Netherlands) equipped with a 20 cm × 75 μm ID fused silica column custom packed with 3 μm 120 Å ReproSil Pur C18 aqua (Dr Maisch GMBH, Ammerbuch-Entringen, Germany). After injection, peptides were trapped at 6 μl/min on a 10 mm × 100 μm ID trap column packed with 5 μm 120 Å ReproSil Pur C18 aqua at 2% buffer B (buffer A: 0.05% formic acid in MQ; buffer B: 80% ACN + 0.05% formic acid in MQ) and separated at 300 nl/min in a 10–40% buffer B gradient in 60 min.

WG and IGD comparison was measured on an FTMS instrument. Eluting peptides were ionized at 1.7 kV in a Nanomate Triversa Chip-based nanospray source using a Triversa LC coupler (Advion, Ithaca, NJ). Intact peptide mass spectra and fragmentation spectra were acquired on a LTQ-FT hybrid mass spectrometer (Thermo Fisher, Bremen, Germany). Intact masses were measured at resolution 50.000 in the ICR cell using a target value of 1 × 10^6^ charges. In parallel, following an FT pre-scan, the top 5 peptide signals (charge-states 2^+^ and higher) were submitted to MS/MS in the linear ion trap (3 amu isolation width, 30 ms activation, 35% normalized activation energy, Q value of 0.25 and a threshold of 5000 counts). Dynamic exclusion was applied with a repeat count of 1 and an exclusion time of 30 s.

Workflow reproducibility was measured on a Q Exactive mass spectrometer (Thermo Fisher, Bremen, Germany) using the same nanoLC separation as described above. Intact masses were measured at resolution 70.000 (at m/z 200) in the orbitrap using an AGC target value of 3 × 10^6^ charges. The top 10 peptide signals (charge-states 2^+^ and higher) were submitted to MS/MS in the HCD cell (4 amu isolation width, 25% normalized collision energy). MS/MS spectra were acquired at resolution 17.500 (at m/z 200) in the orbitrap using an AGC target value of 2 × 10^5^ charges and an underfill ratio of 0.1%.

Dynamic exclusion was applied with a repeat count of 1 and an exclusion time of 30 s.

### Protein identification

#### FTMS data

MS/MS spectra were searched against IPI human 3.62 (83947 entries) (HCT116 cell lysate) or IPI mouse 3.59 (56692 entries) (mouse tumor tissue) using Sequest version 27, rev 12 (Thermo, San Jose, CA, USA). Cysteine carboxamidomethylation and methionine oxidation were treated as variable modifications. Peptide precursor ions were searched with a maximum mass deviation of 10 ppm and fragment ions with a maximum mass deviation of 1 Da. After database searching, Sequest .srf files for each IGD, WG slice pair were imported in Scaffold 3.00.04 (Proteome software, Portland, OR). A protein was considered identified when at least 2 unique peptides were identified in one of the two samples. Peptides were identified with a PeptideProphet [13] probability score >95% and a ProteinProphet [14] probability score >99%. Proteins were (label-free) quantified by spectral counting [15,16] i.e. the sum of all MS/MS spectra for each identified protein (Identified proteins can be found in Additional file 2: Table S2 and Additional file 4: Table S4, and identified peptides in Additional file 3: Table S3 and Additional file 5: Table S5, for human cells and mouse tissue, respectively).

#### Q Exactive data

MS/MS spectra were searched against the Uniprot human complete proteome FASTA file (release January 2013, no fragments; 61608 entries) using MaxQuant 1.3.0.5 [17]. Cysteine carboxamidomethylation was treated as fixed modification and methionine oxidation and N-terminal acetylation as variable modifications. Peptide precursor ions were searched with a maximum mass deviation of 6 ppm and fragment ions with a maximum mass deviation of 20 ppm (default MaxQuant settings). Peptide and protein identifications were filtered at an FDR of 1% using the decoy database strategy. Proteins that could not be differentiated based on MS/MS spectra alone were grouped to protein groups (default MaxQuant settings). Proteins were quantified by spectral counting.

## Abbreviations

CRC: Colorectal cancer; DTT: Dithiothreitol; ID: Identification; IGD: In-gel-digestion; IPI: International protein index; LDS: Lithium dodecyl sulfate; PAGE: Poly acryl amide gel Electrophoresis; SCX: Strong cation exchange; SDS: Sodium dodecyl sulfate; WG: Whole Gel; FFPE: Formalin-fixed paraffin-embedded

## Competing interests

The authors declare they have no competing interests

## Authors’ contributions

SRP designed the experiments, acquired and analysed the data and wrote the manuscript, MOW performed experiments and analysed the data, MdW performed experiments, IdR performed experiments, JCK developed methods and performed experiments, CRJ designed the experiments, supervised the research and wrote the manuscript. All authors read and approved the final manuscript.

## Supplementary Material

Additional file 1: Table 1 Time breakdown of IGD and WG for 10 and 90 gel slices. Click here for file

Additional file 2: Table 2 Identified proteins in HCT116 IGD vs WG. Click here for file

Additional file 3: Table 3 Identified peptides in HCT116 IGD vs WG. Click here for file

Additional file 4: Table 4 Identified proteins in mouse tumor tissue IGD vs WG. Click here for file

Additional file 5: Table 5 Identified peptides in mouse tumor tissue IGD vs WG. Click here for file

Additional file 6: Table 6 Identified proteins in HCT116, reproducibility experiment of WG. Click here for file

Additional file 7: Table 7 Identified peptides in HCT116, reproducibility experiment of WG. Click here for file

Additional file 8: Table 8 Identified proteins in FFPE tumor tissue, reproducibility experiment of WG. Click here for file

Additional file 9: Table 9 Identified peptides in FFPE tumor tissue, reproducibility experiment of WG. Click here for file

## References

[B1] Gao BB Stuart L Feener EP Label-free quantitative analysis of One-dimensional PAGE LC/MS/MS proteome: application on angiotensin II-stimulated smooth muscle cells secretome Mol Cell Proteomics 2008 7 2399 2409 10.1074/mcp.M800104-MCP200 18676994 PMC2596345

[B2] Lawlor K Nazarlan A Lacomis L Tempst P Villanueva J Pathway-based biomarker search by high-throughput proteomics profiling of secretomes J Proteome Res 2009 8 1489 1503 10.1021/pr8008572 19199430

[B3] Albrethsen J Knol JC Piersma SR Pham TV de Wit M Mongera S Carvalho B Verheul HMW Fijneman RJA Meijer GA Jimenez CR Subnuclear proteomics in colorectal cancer. Identification of proteins enriched in the nuclear matrix fraction and regulation in adenoma to carcinoma progression Mol Cell Proteomics 2010 9 988 1005 10.1074/mcp.M900546-MCP200 20089989 PMC2871429

[B4] Piersma SR Fiedler U Span S Lingnau A Pham TV Hoffmann S Kubbutat MHG Jimenez CR Workflow comparison for label-free, quantitative secretome proteomics for cancer biomarker discovery: method evaluation, differential analysis, and verification in serum J Proteome Res 2010 9 1913 1922 10.1021/pr901072h 20085282

[B5] Warmoes M Jaspers JE Pham TV Piersma SR Oudgenoeg G Massink MP Waisfisz Q Rottenberg S Boven E Jonkers J Jimenez CR Proteomics of mouse BRCA1-deficient mammary tumors identifies DNA repair proteins with diagnostic and prognostic value in human breast cancer Mol Cell Proteomics 2012 e-pub ahead of print 10.1074/mcp.M111.013334 PMC3394939 22366898

[B6] Fratantoni SA Piersma SR Jimenez CR Comparison of the performance of two affinity depletion spin filters for quantitative proteomics of CSF: evaluation of sensitivity and reproducibility of CSF analysis using GeLC-MS/MS and spectral counting Proteomics Clinical Applications 2010 4 613 617 10.1002/prca.200900179 21137079

[B7] Shevchenko A Wilm M Vorm O Mann M Mass spectrometric sequencing of proteins silver-stained polyacrylamide gels Anal Chem 1996 68 850 858 10.1021/ac950914h 8779443

[B8] Shevchenko A Tomas H Havlis J Olsen JV Mann M In-gel digestion for mass spectrometric characterization of proteins and proteomes Nat Protoc 2006 1 2856 2860 17406544 10.1038/nprot.2006.468

[B9] Motoyama A Yates JR Multidimensional LC separations in shotgun proteomics Anal Chem 2008 80 7187 7193 10.1021/ac8013669 18826178

[B10] Babu M Krogan NJ Awrey DE Emili A Greenblatt JF Systematic characterization of the protein interaction network and protein complexes in Saccharomyces cerevisiae using tandem affinity purification and mass spectrometry Methods Mol Biol 2009 548 187 207 10.1007/978-1-59745-540-4_11 19521826

[B11] Tang HY Beer LA Speicher DW In-depth analysis of a plasma or serum proteome using a 4D protein profiling method Methods Mol Biol 2011 728 47 67 10.1007/978-1-61779-068-3_3 21468940 PMC3157844

[B12] Tang HY Beer LA Barnhart KT Speicher DW Rapid verification of candidate serological biomarkers using gel-based, label-free multiple reaction monitoring J Proteome Res 2011 10 4005 4017 10.1021/pr2002098 21726088 PMC3166403

[B13] Keller A Nesvizhskii AI Kolker E Aebersold R Empirical statistical model to estimate the accuracy of peptide identifications made by MS/MS and database search Anal Chem 2002 74 5383 5392 10.1021/ac025747h 12403597

[B14] Nesvizhskii AI Keller A Kolker E Aebersold R A statistical model for identifying proteins by tandem mass spectrometry Anal Chem 2003 75 4646 4658 10.1021/ac0341261 14632076

[B15] Liu HB Sadygov RG Yates JR A model for random sampling and estimation of relative protein abundance in shotgun proteomics Anal Chem 2004 76 4193 4201 10.1021/ac0498563 15253663

[B16] Pham TV Piersma SR Warmoes M Jimenez CR On the beta binomial model for analysis of spectral count data in label-free tandem mass spectrometry-based proteomics Bioinformatics 2010 26 363 369 10.1093/bioinformatics/btp677 20007255

[B17] Cox J Mann M MaxQuant enables high peptide identification rates, individualized p.p.b.-range mass accuracies and proteome-wide protein quantification Nat Biotechnol 2008 26 1367 1372 10.1038/nbt.1511 19029910

