# Supplementary material for: Whole gel processing procedure for GeLC-MS/MS based proteomics
Source: Proteome Sci. 2013 Apr 23;11:17. doi: 10.1186/1477-5956-11-17 (PMC3656797; doi:10.1186/1477-5956-11-17)
Supplement: Additional file 1: Table 1 — Time breakdown of IGD and WG for 10 and 90 gel slices. [file 1477-5956-11-17-S1.docx]

Additional Table 1. Time breakdown of IGD and WG for 10 and 90 gel slices

| Day 1  timing steps* | Incubation time (min) | Processing time (min) | | |  |
| --- | --- | --- | --- | --- | --- |
|  |  | IGD 10 | WG 10 | IGD 90 | WG 90 |
| #slices |  | 10 | 10 | 90 | 90 |
| destain ABC | 10 | 2.7 | 0.27 | 24.3 | 0.27 |
| destain ACN/ABC | 10 | 2.7 | 0.27 | 24.3 | 0.27 |
| destain ACN/ABC | 10 | 2.7 | 0.27 | 24.3 | 0.27 |
| reduction DTT | 60 | 2.7 | 0.27 | 24.3 | 0.27 |
| alkylation IAM | 45 | 2.7 | 0.27 | 24.3 | 0.27 |
| washing ABC | 10 | 2.7 | 0.27 | 24.3 | 0.27 |
| washing ABC/ACN | 10 | 2.7 | 0.27 | 24.3 | 0.27 |
| washing ABC/ACN | 10 | 2.7 | 0.27 | 24.3 | 0.27 |
| SUM | 165 | 21.6 | 2.16 | 194.4 | 2.16 |
| slicing time (min) |  | 10 | 10 | 90 | 90 |
| trypsin incubation |  | o/n | o/n | o/n | o/n |
|  |  |  |  |  |  |
| Day 2 |  |  |  |  |  |
| peptide extraction FA | 10 | 2.7 | 2.7 | 24.3 | 24.3 |
| peptide extraction FA-ACN | 10 | 2.7 | 2.7 | 24.3 | 24.3 |
| peptide extraction FA-ACN | 10 | 2.7 | 2.7 | 24.3 | 24.3 |
| SUM | 30 | 8.1 | 8.1 | 72.9 | 72.9 |

*Each step assumes 16.2 sec. per slice (open tube, pipet volume, close tube, -incubate-, open tube, remove volume, close tube); timing from N=2 observations x 10 tubes. The incubation time of each step is shown separately in column 2 and is identical in all procedures and independent of the number of slices. All steps and incubation times can be found in the methods section.

Total time Days 1 and 2

| Time (min) | Day 1 | | | | Day 2 | | | |
| --- | --- | --- | --- | --- | --- | --- | --- | --- |
|  | IGD 10 | WG 10 | IGD 90 | WG 90 | IGD 10 | WG 10 | IGD 90 | WG 90 |
| Incubation time | 165 | 165 | 165 | 165 | 30 | 30 | 30 | 30 |
| gel slicing | 10 | 10 | 90 | 90 | - | - | - | - |
| processing time | 21.6 | 2.16 | 194.4 | 2.16 | 8.1 | 8.1 | 72.9 | 72.9 |
| Total time (min) | 196.6 | 177.16 | 449.4 | 257.16 | 38.1 | 38.1 | 102.9 | 102.9 |
| Total time (Hrs) | 3.3 | 3.0 | 7.5 | 4.3 | 0.6 | 0.6 | 1.7 | 1.7 |
